# A Review of Roles of Uterine Artery Doppler in Pregnancy Complications

**DOI:** 10.3389/fmed.2022.813343

**Published:** 2022-03-03

**Authors:** Yingying Tian, Xiuhua Yang

**Affiliations:** Department of Obstetrics, The First Hospital of China Medical University, Shenyang, China

**Keywords:** uterine artery, pregnancy, recurrent pregnancy loss, preeclampsia, fetal growth restriction, stillbirth, preterm, twin pregnancy

## Abstract

The invasion of trophoblasts into the uterine decidua and decidual vessels is critical for the formation of placenta. The defects of placentation are related to the etiologies of preeclampsia (PE), fetal growth restriction (FGR), and small-for-gestational age (SGA) neonates. It is possible to predict significant vascular events during pregnancy through uterine artery Doppler (UAD). From the implantation stage to the end of pregnancy, detecting changes in uterine and placental blood vessels can provide a favorable diagnostic instrument for pregnancy complications. This review aims to collect literature about the roles of UAD in pregnancy complications. We consider all relevant articles in English from January 1, 1983 to October 30, 2021. Predicting pregnancy complications in advance allows practitioners to carry out timely interventions to avoid or lessen the harm to mothers and neonates. Administering low-dose aspirin daily before 16 weeks of pregnancy can significantly reduce the incidence of pregnancy complications. From early pregnancy to late pregnancy, UAD can combine with other maternal factors, biochemical indicators, and fetal measurement data to identify high-risk population. The identification of high-risk groups can also lessen maternal mortality. Besides, through moderate risk stratification, stringent monitoring for high-risk pregnant women can be implemented, decreasing the incidence of adversities.

## Introduction

The invasion of trophoblasts into the uterine decidua and decidual vessels is critical for the formation of placenta. Nominal blood circulation in the maternal uterine artery is conducive for a healthy intrauterine environment and for sustaining the functionality of the placenta, which ensures the growth of the fetus. It is primarily due to two reasons. First, the maternal blood brings nutrients and carries away the residues. Second, the blood flow in uterine artery affects the oxygen delivered to the maternal-fetal interface. The formation of uteroplacental blood vessels goes through two principal stages. In the first stage occurring before 12 weeks after fertilization, spiral arteries invade the boundary between the decidua and myometrium (1). The second stage occurs from 12 to 16 weeks into the pregnancy, when the spiral arteries invade the interior of the myometrium (2). Accordingly, the two-stage recasting process transforms the narrow myometrial spiral arteries into uterine placental vessels with low resistance. The defects of placentation are related to the etiologies of preeclampsia (PE), fetal growth restriction (FGR), and small-for-gestational age (SGA) neonates (3, 4).

It is possible to predict significant vascular events during pregnancy through uterine artery Doppler (UAD). From the implantation stage to the end of pregnancy, detecting changes in uterine and placental blood vessels can provide a favorable diagnostic instrument for pregnancy complications (5). Quantitative parameters can identify abnormal uterine artery recasting and decreased uterine artery blood flow, and find high-risk pregnant women who are likely to face adverse pregnancy outcomes. Uterine placental vascular impedance gradually declines during angiogenesis and stabilizes after 24 gestational weeks (2). The reduction of placental blood perfusion may occur again in the successive pregnancy (6). The pulsatility index (PI), resistance index (RI), systolic/diastolic (S/D) ratio, and the appearance of an early diastolic notch are common indicators to evaluate uterine artery blood flow (1, 7).

Admittedly, some studies have found that the abnormal uterine artery blood flow was related to the onset of pregnancy complications (2, 8). However, other studies did not confirm this association (9, 10). This review aims to collect literature about the roles of UAD in pregnancy complications. Two independent researchers searched PUBMED for articles with the following medical subject headings (MeSHs): “uterine artery,” “blood flow,” “pregnant,” “recurrent pregnancy loss,” “preeclampsia,” “fetal growth restriction,” “stillbirth,” “preterm,” or “twin pregnancy.” We consider all relevant articles in English from January 1, 1983 to October 30, 2021.

## UAD In a Normal Pregnancy

The uterine artery blood flow begins to increase in the luteal phase and peaks in the window of implantation (11). Past researches have confirmed the reference range of uterine artery parameters in healthy pregnant women from 11 to 41 weeks of pregnancy in different countries (Table 1) (12–21). One study measured the uterine artery blood flow at 24, 28–30, and 34 weeks, respectively (22). It was found that 49% of patients with abnormal blood flow at 24 weeks returned to normal at 34 weeks (22). In a normal pregnancy, there is no significant change in the uterine artery impedance from 24 weeks to the end of pregnancy (23). Besides, maternal race, heart rate, and antihypertensive drug usage influence uterine artery impedance (1). If maternal body mass index (BMI) is too high, the uterine artery PI (UtA-PI) will be less (1). Besides, UtA-PI of the placental side is lower than that of the non-placental side (24).

**Table 1:** Studies on uterine artery Doppler in normal pregnant women.

**Authors**	**Population**	**Gestational weeks**	**Research type**	**Number of subjects**	**Results**
Kurmanavicius et al. 1997 (12)	Swiss	24–42	Cross-sectional study	1675	The 95th percentile of RI fluctuated between 0.56 and 0.61.
Dejthevaporn et al. 2002 (13)	Chinese Taiwanese	22–28	Cross-sectional study	265	Mean PI: < 0.9, 95% CI of PI: < 1.0
Mäkikallio et al. 2004 (14)	Finnish	5–10	Longitudinal study	16	There was no significant change in PI at 5–8 weeks. PI decreased significantly from 8 to 10 weeks.
Gómez et al. 2008 (15)	Spanish	11–41	Cross-Sectional study	620	PI reduced significantly from 11 weeks (mean PI: 1.79; 95th centile: 2.70) to 34 weeks (mean PI: 0.70; 95th centile: 0.99). Between 34 and 41 weeks, the value of PI was relatively stable (mean PI: 0.65; 95th centile: 0.89).
Liao et al. 2009 (16)	Brazilian	11–14; 20–25	Longitudinal prospective study	344	In the first trimester, the 50th and 95th percentile of PI were 1.69 and 2.48, respectively. In the second trimester, the 50th and 95th percentile of PI were 1.03 and 1.57, respectively.
Flo et al. 2011 (17)	Norwegian	22–40	Longitudinal study	53	Mean PI: 0.79-0.56 Mean RI: 0.51-0.40 Mean S/D: 2.0-1.7
Bahlmann et al. 2012 (18)	German	18–42	Cross-sectional study	921	As the pregnancy progresses, reference ranges of PI and RI decreased significantly (PI: 0.89-0.65; RI: 0.45-0.35).
Guedes-Martins et al. 2014 (19)	Portuguese	6–10	Cross-sectional study	312	PI 90th: 4.040 (6 weeks), 3.374 (7 weeks), 3.150 (8 weeks), 2.486 (9 weeks), 2.307 (10 weeks); RI 90th: 1.000 (6 weeks), 0.977 (7 weeks), 0.914 (8 weeks), 0.864 (9 weeks), 0.803 (10 weeks).
Ridding G et al. 2014 (20)	Australian	11–13 +6	Prospective study	298	Gestational age and the mean PI was negatively correlated.
Stridsklev et al. 2017 (21)	Norwegian	8–24	Longitudinal study	124	The 97.5th percentile of PI varied from 1.03 to 4.07.

*RI, resistance index; CI, confidence interval; PI, pulsatility index; S/D, systolic/diastolic velocity*.

“Notching” is a relatively common characteristic that appears during the early stages of 46–64% of normal pregnancies (1). A diastolic notch is a reduction in the maximum diastolic blood flow by at least 50 cm/s after 20 gestational weeks (25). However, most reports adopt subjective diagnostic criteria. Like UtA-PI, the incidence of “notch” decreases after 24 weeks of pregnancy and remains stable. Continuous early diastolic notch is a manifestation of abnormal uterine vascular tension, and poor placentation may increase uterine artery impedance (26). Considering the low repeatability of uterine artery notch, UtA-PI is preferred as an indicator of vascular impedance with its objective detection.

Transabdominal or transvaginal ultrasound can both detect UAD. During the testing process, UAD needs to be checked according to the standard procedure to obtain accurate results. Clinically, transabdominal ultrasonography is more acceptable, primarily because it is non-invasive with excellent repeatability. A prospective study compared the UtA-PI values using transabdominal or transvaginal ultrasound at 11–13 + 6 weeks of gestation (27). The results revealed that transabdominal ultrasound showed lower UtA-PI values (*P* < 0.05) (27).

## UAD In Recurrent Pregnancy Loss (RPL)

RPL refers to two or more pregnancy losses before 20–24 weeks of pregnancy (28). It is a critical reproductive disease, with an incidence rate of 5% in couples of childbearing age (29). The causes of RPL include uterine malformations, chromosomal abnormalities, immune abnormalities, endocrine or metabolic diseases, thrombophilia, and genetic factors (30, 31). The etiology of 50% of RPL patients is unclear (32). There is evidence that the occurrence of RPL may be related to the decrease of blood perfusion in the endometrium and myometrium (8, 33–35).

Patients with unexplained RPL (uRPL) had increased uterine artery resistance and decreased sub-endometrial blood flow (36). UAD is an indicator of the state of uterine blood circulation in uRPL patients (8). One study tested 214 pregnant women for uterine artery blood flow by transvaginal color Doppler ultrasound at 5–12 weeks of pregnancy (37). The study formed two groups based on whether these women could continue their pregnancies at 20 weeks (37). The results showed that the S/D ratio of uterine artery in pregnant women who could continue the pregnancies was significantly lower than that of the abortion group (4.3 *vs*. 5.3; *P* = 0.0001) (37). It suggests that the change of blood flow in the uterine vascular bed could help predict embryo loss in early pregnancy (37). Several studies have confirmed that the mid-cycle uterine artery blood flow was markedly reduced in RPL females (33, 38). However, studies also reported that no significant difference was present in PI, RI or S/D ratio of the uterine artery between RPL patients and healthy pregnant women at 18–23 days of the menstrual cycle (10). Therefore, a randomized controlled study with large samples is required for further analysis.

Antinuclear antibodies (ANA) is a crowd of autoantibodies against nuclear and cytoplasmic antigens. There is a higher likelihood for ANA to occur in RPL patients compared with normal pregnant women (39). ANA positive patients had a higher probability of poor outcomes among women with *in vitro* fertilization (IVF), indicating the harmful effect of ANA on the development of oocytes and embryos (40). One study measured UtA-PI in 26 uRPL patients and 26 normal women during the follicular and mid-luteal phase, respectively (41). In both test periods, UtA-PI of ANA positive RPL patients was significantly higher than that of ANA negative RPL patients and healthy women (41). ANA is likely to change the hemodynamics of the uterine artery by affecting the intensity and impedance of uterine artery, resulting in uRPL (41). A previous study also confirmed these findings (8). However, ANA results did not have a relationship with UtA-PI in normal pregnancies (8). Besides, UtA-PI of ANA positive uRPL patients was also higher than that of healthy pregnant women in early pregnancy (42).

Over the recent years, researchers have begun to pay attention to the uterine radial artery (URA), which is the lower branch of the uterine artery passing through the endometrium. As a result, URA can better reflect the blood flow of the endometrium (43). Compared with UtA, URA can better represent the blood flow transmitted to the fetus in the first trimester (44). After 5 weeks of gestation, URA-RI decreases significantly, which may represent the recasting of blood vessels at the maternal-fetal interface during placentation (44). Meanwhile, after 10 gestational weeks, there is a marked reduction of UtA-RI, reflecting the total uterine blood flow related to the uterine enlargement (44). A prospective study examined the blood flow of URA in 33 RPL patients and 47 normal pregnant women at 5–7 weeks of gestation (45). It was found that URA-RI of RPL patients was significantly higher than that of normal controls (*P* < 0.05) (45). In addition, the proportion of peripheral blood natural killer (pbNK) cells in RPL patients was positively correlated with URA-RI (*P* < 0.05) (45). Another retrospective analysis involved 139 patients with RPL and thrombophilia (5). URA-RI at 8 gestational weeks was notably higher in patients who had an abortion in the index pregnancy than that of pregnant women who delivered live newborns (5). After covariates such as maternal age, BMI, and the number of abortions were adjusted, the risk of abortion was raised by 18.7% when URA-RI increased by 0.1 at 8 weeks of pregnancy (5). These studies emphasized the importance of detecting the blood flow of UtA and URA in RPL patients.

## UAD and PE/FGR

PE is a leading cause of maternal and perinatal death in both developed and under-developed countries. It is the cause of nearly 10–15% of maternal deaths (46). Pregnant women with PE face an elevated risk of cardiovascular disease in the future (47). FGR is often associated with PE, which means that the predicted fetal weight is below the 10th percentile due to poor placental formation, or even if the ultrasound is normal, the predicted fetal weight is below the third percentile (48, 49). FGR is the primary cause of perinatal morbidity and mortality. At present, it is challenging to distinguish FGR caused by decreased placentation from healthy small infants.

In the past decades, numerous studies have revealed reliable and safe indicators to predict PE. PE or FGR is more likely to occur in pregnant women with alternations in pregnancy-associated plasma protein-A (PAPP-A), placental growth factor (PlGF), and soluble fms-like tyrosine kinase-1 (sFlt-1) levels (50, 51). However, it is challenging to carry out these expensive tests in economically under-developed areas. Moreover, countries that spend less on health care may not increase additional investment in research to change current guidelines. Under-developed countries should seek advanced means to adjust the current strategy, benefiting more people (52). UAD is such an economical approach, which may provide a novel method to forecast PE and FGR in under-developed countries.

### Studies of UAD in the First Trimester

Recently, researchers have increasingly studied the application of UAD in the first trimester (Table 2). A study considered uterine artery mean PI > 2.35 as the cut-off value for predicting PE or FGR among low-risk pregnant women at 11–14 weeks of gestation (55). The predictive sensitivity of UtA-PI greater than the 95th percentile was low (12–27%); however, the specificity was very high (95%) (55). According to this article (55), UAD at 11–14 weeks can confirm most pregnant women who will have PE and/or FGR. A prospective research involved 120 Caucasian pregnant women with high-risk factors for PE at 11–14 gestational weeks (70). Accordingly, the predictive sensitivity of UtA-PI for PE was 61.5, and the specificity was 63.8% (70). Adding the bilateral notch improved the sensitivity (65.4%) and specificity (66%) (70). Therefore, the authors believed that UAD was a useful non-invasive method for predicting PE, especially in areas with poor economic conditions and unable to afford other detection items (70). However, in a separate study, UtA-PI greater than the 95th percentile had only 23.9% sensitivity for predicting PE among 999 low-risk pregnant women in early pregnancy (58). In the first trimester, the application of UAD alone for predicting gestational hypertension is limited, and other indices should be considered to combine with UAD (58). A prospective study examined the uterine artery blood flow in 405 pregnant women at 11–13 + 6 weeks (68). The experiment did not find any significant difference in UtA-PI and the presence of uterine artery notch between pregnant women with PE and those without PE (68). Besides, a large sample study of 8,061 pregnant women revealed that the lowest UtA-PI of pregnant women with PE was significantly higher than that with normal pregnancy outcomes (71). Meanwhile, some experts highlighted that a single value of UtA-PI can not precisely reflect the uterine artery resistance, so they recommended using the mean PI in multiples of the median (MoM) after adjusting the basic maternal data (72). Another study measured the uterine artery blood flow in 3,058 pregnant women at 11–14 weeks (60). Follow-ups confirmed that 57 of them had full-term PE and 33 experienced preterm PE (60). UtA-RI of preterm PE patients (mean RI: 0.79) was significantly higher than that of patients with normal pregnancy outcomes (mean RI: 0.70) or full-term PE (mean RI: 0.72; *P* < 0.05) (60). However, there was no significant difference in UtA-RI and the appearance of bilateral notch between full-term PE and normal pregnant women (60).

**Table 2:** Studies on uterine artery Doppler (UAD) in preeclampsia (PE) and fetal growth restriction (FGR).

**Authors**	**Population**	**Gestational weeks**	**Research type**	**Number of subjects**	**Results**	**Conclusions**
Kurdi W et al. 1998 (53)	British	19–21	Prospective study	946	The OR of women with diastolic notches to have PE was 12.8, and the OR of developing PE requiring termination of pregnancy before 37 weeks was 52.6.	Patients with increased uterine artery resistance had a higher tendency to experience pregnancy complications, particularly the ones needed to terminate the pregnancy before 37 weeks.
Papageorghiou AT et al. 2001 (54)	British	22–24	Prospective study	7,851	The sensitivity of PI greater than the 95th percentile to predict PE with FGR, PE without FGR, FGR without PE, PE with or without FGR, FGR with or without PE were 69, 24, 13, 41, and 16%, respectively.	Uterine artery Doppler at 23 gestational weeks could detect most severe PE and/or FGR patients.
Martin AM et al. 2001 (55)	British	11–14	Prospective study	3,045	The sensitivity of PI greater than 2.35 to predict PE (with or without FGR) and FGR (without PE) was 27 and 11.7%, respectively. The sensitivities of PE and FGR that requires terminating the pregnancy 32 weeks ago was 60.0 and 27.8%, respectively.	UAD betwen 11 and 14 weeks confirms most patients with severe PE and/or FGR.
Phupong V et al. 2003 (56)	Thai people	22–28	Prospective study	322	The sensitivity, specificity, PPV, and NPV to predict PE were 36.8, 83.2, 12.1, and 95.5%, respectively; and those for SGA neonates were 67, 82.9, 6.9, and 99.2%, respectively.	It is likely that pregnant women with diastolic notch would have PE and SGA neonates.
Vainio M et al. 2005 (57)	Finnish	12–14	Prospective trial	120	As the pregnancy progresses, the sensitivity, specificity, PPV and NPV of bilateral notches to predict gestational hypertension ranged from 91–35, 41–94, 7–70, and 86–97%.	The detection of diastolic notches between 12 and 14 weeks could be an indicator of gestational hypertension in high-risk pregnant females.
Gómez O et al. 2005 (58)	Spanish	11–14	Prospective study	999	In comparison with healthy pregnancies, pregnant women with complications had higher PI and an elevated incidence of bilateral notch (*P* < 0.05).	UtA-PI in gestational hypertension patients increased significantly during early pregnancy. Nevertheless, the clinical significance of only monitoring the uterine artery was relatively small in the low-risk population in the first trimester.
Ricardo S et al. 2008 (59)	Brazilian	22–24	Prospective study	1,057	The RRs of PI > 1.55 for PE and FGR were 7.3 and 3.9.	UAD at 22–24 weeks can be used to identify pregnant women with complications due to poor placental function.
Melchiorre K et al. 2008 (60)	British	11–14	Prospective study	3,058	UtA-RI of preterm PE patients was significantly higher than that of healthy pregnant females or full-term PE women (*P* < 0.05). There was no significant difference in UtA-RI and the presence of diastolic notch between full-term PE patients and normal pregnancies.	Indeed, UAD in early pregnancy was related to preterm PE. However, this study did not support adding UAD to routine prenatal examination.
Plasencia W et al. 2008 (61)	British	11–13 + 6 and 21–24+6	Prospective study	3,107	Maternal characteristics, UtA-PI at 11–13 + 6 weeks, and the change of UtA-PI from 11–13 + 6 weeks to 21–24 + 6 weeks were remarkable independent indicators to predict PE.	Useful monitoring for PE could be realized by the Doppler detection of UtA-PI at 11 to 13 + 6 weeks and the alternation in PI between 11 and 13 + 6 and 21 to 24 + 6 weeks.
Melchiorre K et al. 2009 (62)	British	11–14	Prospective study	3,010	Compared with normal pregnancies, UtA-RI and the presence of diastolic notch in the first trimester among pregnant women with SGA newborns were significantly higher.	There was a significant correlation between UtA-RI in early pregnancy and the following SGA neonates.
Ghi T et al. 2010 (63)	Italian	20–22; 26–28	Prospective study	208	Compared with pregnant women with nominal uterine artery blood flow at 22–22 weeks and those with normal uterine artery parameters at 26–28 weeks, patients with persistent abnormal UAD were more prone to PE, SGA neonates and admission to NICU.	For low-risk primiparas with abnormal UAD in the second trimester, the probability of pregnancy complications increased if UAD at 26–28 weeks was persistently abnormal.
Maroni E et al. 2011 (64)	Italians	34	Prospective study	132	Pregnant women with elevated UtA-PI exhibited an earlier gestational week of delivery, having lighter fetal weight and a higher proportion of SGA fetus (*P* < 0.05).	Elevated UtA-PI at 34 weeks was an independent indicator of a higher incidence of having an SGA infant.
Lai J et al. 2013 (65)	British	30–33	Prospective study	4,294	By combining maternal factors and UtA-PI, 70.3% intermediate- (34–37 weeks) PE and 54.6% late-PE (>38 weeks) could be found.	Combination of maternal factors and UtA-PI at 30–33 weeks could authentically recognize females with a higher probability of PE.
Barati M et al. 2014 (66)	Iranian	16–22	Cross-sectional study	379	The sensitivity, specificity, NPV, PPV of UtA-PI greater than 1.45 for predicting PE were 95.%, 79, 98.9, and 88.2%, respectively. For predicting SGA, the corresponding numbers were 96.5, 57, 99.2, and 23.5%, respectively.	UAD detection at 16–22 weeks could be a suitable instrument to predict PE and SGA.
Parry S et al. 2017 (67)	American	16–22 + 6	Prospective study	8,024	The nominal thresholds of uterine artery indices were related to SGA. However, they had low PPVs (< 15%) and unsatisfactory AUCs (0.5–0.6).	UAD measurement in the early second trimester was not an effective means to predict SGA infants.
Prakansamut N et al. 2019 (68)	Thai people	11–13 + 6	Prospective study	405	A significant difference was not present in UtA-PI and the presence of uterine artery notch between PE patients and those without PE.	UtA-PI in the first trimester is not an independent predictor for PE.
Običan SG et al. 2020 (69)	American	24–36	Nested case control study	200	Patients with diastolic notch in the left uterine artery and PI greater than the 95th percentile faced an elevated risk of pregnancy complications.	UAD had a moderate predictive effect when predicting adverse maternal and fetal outcomes.

*OR, odds ratio; PI, pulsatility index; PPV, positive predictive value; NPV, negative predictive value; SGA, small for gestational age; RI, resistance index; RR, relative risk; UtA, uterine artery; NICU, neonatal intensive care unit; AUC, areas under receiver operating characteristic curves*.

A meta-analysis explored the predictive effect of UAD on PE and FGR in early pregnancy (3). The authors assessed 55,974 females in 18 studies, and 15 of them were performed among low-risk pregnant females (3). In early pregnancy, the predictive sensitivity of anomalous UAD on PE and early-onset PE was 26.4 and 47.8%, respectively (3). Moreover, 15.4% of FGR could be forecasted, and the prediction sensitivity of early-onset FGR was 39.2% (3). Similarly, another meta-analysis of 298,329 primiparas from 76 studies suggested that the prediction of UAD on adverse pregnancy outcomes was limited in the first and early stage of the second trimester of pregnancy (73). As an independent index, UAD has a medium predictive effect in the low-risk population in the first trimester, around 40–70% (1). The prediction ability for early-onset PE can reach more than 90% using the multi-parameter prediction model, including UtA-PI, maternal characteristics, and biochemical indices (1).

### Studies of UAD in the Second Trimester

It could be beneficial to screen UAD in a high-risk population in the second trimester. Reportedly, at 23–24 weeks, the detection rate of abnormal uterine artery blood flow to PE was 45% in high-risk pregnant women (2). After 20 gestational weeks, UtA-RI greater than the 90th percentile is a potential indicator of gestational hypertension or FGR for pregnant women with a moderate risk of PE (74). For high-risk multiparous pregnant females, most pregnant women who have a bilateral notch with RI ≥ 0.55 and a unilateral notch with RI ≥ 0.65 at 20 weeks will experience adverse pregnancy outcomes due to poor placentation (75). Compared with clinical high-risk factors, UAD has a better predictive effect on PE and SGA neonates among high-risk females at 22–24 weeks (76).

Uterine artery monitoring among low-risk pregnant women is also helpful in the second trimester (59, 77, 78). A UtA-PI greater than 1.45 in the second trimester was a critical indicator in predicting PE in low-risk pregnant women (66). The detection of uterine artery Doppler waveform and the appearance of a notch in the second trimester can be used to monitor pregnant women who may have adverse pregnancy outcomes (66). A retrospective study analyzed the uterine artery blood flow in 1,472 pregnant women from 19 to 22 gestational weeks (79). Pregnant women with high UtA-RI, PI or the appearance of a diastolic notch were more likely to develop FGR, SGA, lower Apgar score at birth, increased cesarean section rate, spontaneous preterm birth (PTB), and placental abruption (*P* < 0.05) (79). The appearance of diastolic notch markedly promoted the incidence of severe PE, HELLP syndrome and oligohydramnios (79). It has been reported that the appearance of uterine artery notch show impaired endothelial function (80, 81). The uterine artery testing of 30,639 unselected pregnant women revealed that UAD at 22–24 weeks could predict most early-onset PE rather than late-onset PE (4).

It is also necessary to detect the presence of uterine artery notch in the second trimester. The detection of UAD in 652 pregnant women at 12–16 weeks of pregnancy revealed that the existence of bilateral notches elevated the incidence of PE, spontaneous PTB, and SGA newborns (78). Another study that included 1,536 pregnant women at 16–23 gestational weeks revealed that the positive likelihood ratios of mean notch depth index (mNDI) and mean pulsatility index (mPI) for forecasting early-onset PE had a medium predictive effect (82). It suggests that mNDI or mPI in the second trimester can help identify high-risk groups that might have early-onset PE (82). Besides, other studies have shown that the combination of NDI and UtA-PI can predict different adverse pregnancy outcomes, such as placental abruption, FGR, stillbirth, and spontaneous PTB before 32 weeks (83, 84).

A few studies with large sample numbers have obtained valuable results. A multicenter analysis detected UtA-PI in 7,851 pregnant women at 23 gestational weeks (54). The sensitivity of UtA-PI greater than the 95th percentile for predicting PE (with or without FGR) and FGR (with or without PE) was 41 and 16%, respectively (54). Moreover, for pregnant women who terminated their pregnancies before 32 weeks due to PE with FGR, PE without FGR, or FGR without PE, the sensitivity of UtA-PI was 93, 80, and 56%, respectively (54). In a retrospective study including 23,894 pregnancies at 19–24 gestational weeks, UtA-PI alone identified 25–77% SGA (less than the 5th quantile) who had deliveries at different gestational weeks with a false positive rate (FPR) of 10% (85). In a meta-analysis with 79,547 PE patients and 41,131 FGR pregnancies, the positive likelihood ratio of increased UtA-PI and the presence of notch to predict PE was the highest (21 in a high-risk population and 7.5 in a low-risk population) (86).

However, the outcomes of a study performed in low-risk primiparas suggested that UAD in the second trimester had a slight predictive effect on PE (9). Similarly, another analysis examined the uterine artery blood flow in 2,489 low-risk pregnant women at 22 weeks (87). The results of the logistic regression model showed that the predictive sensitivity of PE and SGA were only 44.8% and 28.1%, respectively (87). Accordingly, UAD was not beneficial to predict PE and FGR in the low-risk population at 22 weeks (87). Moreover, scholars collected 8,024 primiparas and tested their uterine artery blood flow from 16 to 22 + 6 weeks (67). The typical thresholds of uterine artery parameters were related to SGA (67). However, all positive predictive values (PPV) were less than 15%, and the areas under receiver operating characteristic curves (AUCs) was 0.5–0.6 (67). The study indicated that UAD in the early second trimester executed weak functionality on predicting SGA neonates (67). The contradiction in the findings may be related to the heterogeneity of research objects, different gestational weeks, and the diverse reference range of uterine artery parameters.

### Studies of UAD in the Third Trimester

Some studies have reported that the continually increasing resistance of blood flow in the uterine artery in PE, FGR, or SGA patients during the third trimester may lead to many adverse pregnancy outcomes (88–96). These adversities include stillbirth, cesarean section due to fetal distress, and decreased PH of the umbilical cord (88–96). A comparative analysis of the pregnancy outcomes between normotensive patients with increased UtA-PI and pregnant women with normal uterine artery blood flow at 34 weeks of gestation revealed that pregnant women with increased UtA-PI delivered at earlier gestational weeks, having lower fetal weight, and a higher incidence of SGA infants (*P* < 0.05) (64). Admittedly, the predictive effect of UtA-PI on late-onset PE was lower than that of early-onset PE. However, recent findings suggest that the predictive effectiveness of late-onset PE by UtA-PI gradually increases in the third trimester (97, 98). In addition, another study found that UtA-PI of full-term PE patients increased significantly only from 33 weeks, indicating the necessity to monitor uterine artery blood flow in the third trimester (99). The increase of UtA-PI in full-term PE patients in the third trimester may be the result of vasoconstriction in uterine placental circulation just before the onset of the disease, instead of the defective placentation at early pregnancy. A research analyzed UAD in 86 late-onset PE patients (>34 weeks) (100). It was found that the abnormalities of UAD were associated with severe PE, late spontaneous PTB, SGA, and neonatal intensive care unit (NICU) admission (*P* < 0.05) (100). Accordingly, UAD was associated with placental apoptosis, indicating that placental dysplasia caused some late-onset PE cases (100).

Remarkably, regarding screening patients with late-onset placental-related diseases, the detection of UtA-PI in the third trimester performed better than that in the first trimester, since they had new-onset increased UtA-PI in the third trimester instead of the first trimester (101). The plausible reason is that increased UtA-PI in the third trimester is not related to the change of trophoblasts. Further, there was no significant change in the uterine artery blood flow between patients with abnormal placental implantation and pregnant women with normal placental implantation (102, 103). In addition, the changes of the uterine artery in the third trimester could also be related to cardiac output, physiological changes of the blood system, and systemic vascular resistance (104). There is a hypothesis that increased UtA-PI in the third trimester may cause subsequent PE, which is related to the change of the maternal blood system. Besides, the report that PE is related to cardiac recasting and significant cardiac damage supports the theory (105). Reportedly, the maternal cardiovascular function determines whether a pregnant woman will have PE (106). Indeed, there is evidence that late-onset increased UtA-PI is associated with abnormal maternal hemodynamics (106). However, a study found that placental hypo-perfusion occurred in 66.7% of placentas in patients with late-onset SGA neonates (107). The abnormal development of distal villous caused nearly 25 of placental hypo-perfusion, and 50% was due to the blockage of blood vessels, resulting in elevated UtA-PI during the third trimester (108).

### Sequential UAD Screening

A study performed sequential detection of UAD in 870 pregnant women at 11–14 and 19–22 weeks, respectively (109). Based on the outcomes, pregnant women with persistently elevated UtA-PI faced the greatest risk for gestational hypertension and FGR (109). The changes in the uterine artery blood flow from the first trimester to the second trimester were related to gestational hypertension and FGR (109). A separate sequential analysis detected UtA-PI in 3,107 pregnant women at 11–13 + 6 weeks and 21–24 + 6 weeks (61). Multiple regression analysis showed that maternal factors, UtA-PI at 11–13 + 6 weeks, and the alternation of UtA-PI from 11–13 + 6 weeks to 21–24 + 6 weeks were independent factors to predict PE (61). Compared with PE patients, UtA-PI was significantly less in normal pregnant women from 11–13 + 6 weeks to 21–24 + 6 weeks (61). The continuous monitoring could re-evaluate 75% of patients who were assessed as the high-risk population in 11–13 + 6 weeks as low-risk patients at 21–24 + 6 weeks to avoid the anxiety of these pregnant women and reduce the workload of physicians (61). Moreover, practitioners chose 243 unselected pregnant women to test for uterine artery notches at 12–14 and 22–24 gestational weeks (110). They found that patients without a notch or early disappearance of notches rarely had adverse pregnancy outcomes due to poor placentation (110). A retrospectively study reviewed the results of UtA-PI in 5,887 pregnant women in the second and third trimesters (111). Patients with continuously increased UtA-PI were more likely to develop gestational hypertension compared to those without (*P* < 0.05) (111). The results indicate an association between the continuous deterioration of uterine artery blood flow and gestational hypertension, independent of UtA-PI recorded in the second trimester (111).

### The Combination of UAD and Other Factors

UtA-PI can be combined with maternal factors, biophysical and biochemical indicators at 11–13 + 6 weeks to predict PE and SGA (112). Combining maternal factors, UtA-PI, mean arterial pressure (MAP), and PlGF can forecast 75 of preterm PE and 47% of full-term PE (113). Between 19 and 24 gestational weeks, combination of maternal factors, PI, and PlGF could predict 99, 85, and 46% of PE before 32 weeks, before 37 weeks, and after 37 weeks, respectively (114). The combination of UtA-PI and biochemical indices in the second trimester exhibited higher sensitivity than the prediction by medical history alone (114). At 30–34 weeks of gestation, combined detection with maternal factors, UtA-PI, MAP, PlGF, and sFlt-1 identified 99 of preterm PE and 49% of full-term PE (97). Accordingly, UtA-PI combined with maternal factors and biochemical indicators in the third trimester could find almost all preterm PE and half full-term PE with 5% of FPR (97). However, an empirical analysis from two British hospitals showed that UtA-PI at 30–34 weeks of gestation could predict 79 premature PE and 42% full-term PE (115). Moreover, combining biochemical results and MAP in early and/or middle pregnancy with UtA-PI did not have a significant improvement in predicting preterm PE and full-term PE (115).

According to some studies, the combination of maternal variables, abnormal UAD, and low PAPP-A levels in the first trimester may help predict PE and SGA in the third trimester (116, 117). Combining serum Split and Hairy-related Protein 1 (SHARP1), inhibin-A expressions, sFlt-1/PlGF ratio, and UAD could also enhance the screening efficacy for predicting PE (68, 118, 119). A recent prospective research studied 378 pregnant women with high-risk factors for early-onset PE by adopting the multivariate prediction model of early pregnancy (120). The sensitivity of predicting SGA by combining UtA-PI, PlGF, and sFlt-1 was low in the third trimester (120). However, a high negative predictive value can alleviate the anxiety of pregnant women, avoid unnecessary medical intervention, and formulate individualized treatment plans for high-risk groups (120). The contradicting results of different studies may be due to the diverse definitions of PE and FGR, different methods of Doppler detection (pulsed, color or continuous wave), distinct diagnostic criteria of positive cases, different subjects, and the usage of aspirin and/or low molecular weight heparin (LMWH).

## UAD and Stillbirth

Poor placentation could likely be the etiology of stillbirth. Reasonable prenatal intervention and timely treatment may lessen the incidence of stillbirth (121). In the first trimester, 61% of stillbirths due to poor placentation could be identified by combining maternal factors, UtA-PI, fetal ductus venosus PI for veins, and PlGF, with a FPR of 10% (122). In contrast, the sensitivity of UAD alone in predicting stillbirth in the first trimester was only 14.5% (3). A study included 15,835 pregnant women in the second trimester, and 144 experienced stillbirths (123). Pregnant women with uterine artery parameters greater than the 90th percentile had a 7-fold higher risk of stillbirth than those with uterine artery parameters below the 90th percentile (123). Besides, another prospective study with a large sample size including 70,003 pregnant women also showed that a combination of maternal factors, UtA-PI and fetal measurement parameters in the second trimester could predict 55 of stillbirths, of which 75% were caused by defective placentation (124). Reportedly, UAD in the second trimester effectively predicts early stillbirths caused by PE, placental abruption, or SGA, instead of late stillbirth without PE, placental abruption, or SGA (125). Additionally, a study of 30,519 pregnant women showed that higher UtA-PI in the second trimester had a high predictive sensitivity for stillbirth occurred before 32 weeks caused by placental issues (126). UtA-PI combined with cerebroplacental ratio (CPR) and estimated fetal weight (EFW) could predict stillbirth in the third trimester with a sensitivity of 66.7%, specificity of 92.1% (95).

## UAD and Spontaneous PTB

Spontaneous PTB refers to delivery before 37 weeks of pregnancy with a worldwide incidence of around 11% (127). Reportedly, abnormal placental formation and decreased uterine artery blood flow were related to spontaneous PTB (128, 129). UtA-PI in the first trimester could not predict spontaneous PTB effectively (130). Regarding the relationship between UtA-PI and spontaneous PTB in the second trimester, UtA-PI was higher in patients with deliveries before 33 weeks when compared with those who had childbirth at and after 33 weeks (131). The increase of UtA-PI in patients with spontaneous PTB may be the result of decreased placental function, since histopathological findings have revealed that the remodeling of uterine spiral arteries in these patients was insufficient (128). However, this study showed that UAD did not predict better than maternal medical history and clinical features (131). Another study tested 4,521 pregnant women for UAD at 18–22 weeks of pregnancy (132). UtA-RI of spontaneous PTB patients was significantly higher than that in full-term pregnant women (1.12 *vs*. 1.04; *P* < 0.05). Patients with increased UtA-RI had an elevated risk of spontaneous PTB (OR 2.9 per unit; 95% CI 2.4–3.9) (132). Nearly one-third of pregnant women with bilateral diastolic notches had spontaneous PTB (132). The analysis pointed out that UtA-RI in the second trimester was a weak predictive index of spontaneous PTB (132). A retrospective study compared UtA-RI measured during the second trimester between 234 spontaneous PTB patients and 5,472 full-term pregnant females (133). There was no significant difference in UtA-RI between the two groups (133).

## UAD and Twin Pregnancy

In the first (134, 135) and second trimesters (136), the UtA-PI of twin pregnant women was significantly lower than that of a single pregnant female. Similarly, in the second trimester, the UtA-RI of twins was markedly lower than that of singletons (137). The overall reduced UtA-PI and RI may represent an increased placental implantation area and distinct changes in maternal hemodynamics in twin pregnancy. Another study confirmed that the UtA-PI of twin pregnancies was lower than that of a single pregnancy from 17 to 38 weeks of gestation (138). According to this study, it is necessary to establish the normal range of uterine artery parameters for twin pregnant women to distinguish between high-risk pregnant women and low-risk population (138). It has also been reported that the UtA-PI of dichorionic twins was lower than that of monochorionic twins in the first (134) and the second trimesters (139). However, other studies have not confirmed the result. In fact, some studies reported that there were no differences in the UtA-PI between monochorionic and dichorionic pregnancies in the first trimester (135) and the second trimesters (140). This shows that the total size of placenta determines the blood flow of uterine artery, rather than the genetic or hormonal factors related to chorionicity (141).

Higher UtA-PI in early pregnancy was related to PE and SGA in the index pregnancy, suggesting that UAD in the first trimester could predict adverse pregnancy outcomes in twin pregnancy (135). The predictive validity of UtA-RI for forecasting gestational hypertension and/or PE was frustratingly low in twin pregnancies from 20 to 24 weeks (137). In another study performed in second trimester, the sensitivity of mean, lowest and highest UtA-PI for predicting PE was 35, 29, and 27%, respectively (136). Still, the sensitivity was not very high. However, the authors believed that the screening of uterine arteries had great clinical significance considering the increased probability of PE in twins (136). In a study that included 406 healthy twin pregnant women in the second trimester, the authors found that the sensitivity of UAD for predicting adverse complications was lower than that of singletons (139). Likewise, a retrospective study comprising 256 dichorionic twin pregnant women has been conducted (142). Accordingly, the sensitivity of UAD in predicting adverse pregnancy outcomes was low despite the application of the reference interval for twins (142). The negative predictive value of UAD in twins was lower than that in singletons, suggesting that adverse pregnancy outcomes could still occur despite the existence of normal uterine artery blood flow in twins (142). It indicates that the etiologies of PE and FGR in twins may have other causes that are not associated with placental dysfunction (142). Besides, there is no consensus on whether low-dose aspirin can prevent PE in high-risk twins before 16 weeks of pregnancy (143). Thus, the application of UAD in twin pregnant women seems to be limited.

## Clinical Implications of Screening UAD

Predicting pregnancy complications in advance allows practitioners to prevent and carry out timely interventions to avoid or lessen the harm to mothers and neonates. Administering low-dose aspirin daily before 16 weeks of pregnancy can significantly reduce the incidence of pregnancy complications, including spontaneous PTB, FGR, PE, and perinatal death (144). The Cochrane review, published in 2019 (145), found that in pregnant women with a risk of developing PE, the use of low-dose aspirin reduced the risk of PE, spontaneous PTB, and perinatal death by 18, 9, and 14%, respectively. Noticeably, aspirin slightly increases the incidence of postpartum hemorrhage and placental abruption (145). Besides, preventive treatment using aspirin in pregnant women with abnormal uterine artery blood flow in the second trimester can significantly reduce the incidence of PE (146). However, another meta-analysis indicated that commencing aspirin treatment in the second trimester has minimal effect in reducing the risk of subsequent PE (147). A recent report revealed that aspirin could reduce the risk of preterm PE, rather than full-term PE, only when it was prescribed from 16 weeks' gestation at a daily dosage of more than 100 mg (148). Although the preventive effect of aspirin in the second trimester is not clear, the risk stratification of pregnant women in the second and third trimesters can strengthen the monitoring of the high-risk population and formulate an individualized mode and timing of delivery. Additionally, the application of LMWH can improve uterine artery blood flow and help obtain good pregnancy outcomes in uRPL patients with increased URA-RI (45). Reportedly, alternative therapeutic approaches comprising nitric oxide, sildenafil, omega 3, and vitamin E can also increase uterine artery blood flow in pregnancy complications (36, 149–151).

Under modern clinical settings, every pregnant woman has a routine ultrasound examination in the first trimester in most parts of the world. In the U.K., additional screening of UAD costs only 18–25 pounds and an additional 5 min (152, 153). The FPR of UAD is relatively low, so it will not increase the anxiety of pregnant women. Besides, the inspection of uterine artery blood flow is a relatively fast and low-cost item. Therefore, it can be regarded as a routine monitoring procedure in obstetrical ultrasound.

## Conclusions

According to the published studies, UAD can be used to predict pregnancy complications such as RPL, PE, FGR, stillbirth, and spontaneous PTB. For early-onset PE/FGR caused by poor placentation, the accuracy of prediction was more than 90% when combining UAD with other biochemical indices. Monitoring UAD among high-risk pregnant women in the third trimester can also help predict late-onset PE/FGR and guide the mode of delivery. A critical aspect of obstetrical work is to seek high-risk groups that may have adverse pregnancy outcomes early. Primiparas have no obstetric history, so it is hard to assess pregnancy risk. Presently, limited medical resources are distributed among all pregnant women, but not among those most likely to have adverse pregnancy results. As a non-invasive examination, UAD can help clinical practitioners identify high-risk groups and implement specific detection and prevention measures. From early pregnancy to late pregnancy, UAD can combine with other maternal factors, biochemical indicators, and fetal measurement data to identify high-risk population. The identification of high-risk population can also lessen maternal mortality. Besides, through moderate risk stratification, stringent monitoring for high-risk pregnant women can be implemented, decreasing the incidence of adversities.

However, inconsistencies in detection methods affect the clinical application of UAD. A unified standard is lacking in terms of a clinically acceptable gestational age at which assessment should be made, the equipment used, and whether to use a diastolic notch or more objective indicators, such as impedance and velocity indices. Besides, the blood circulation in the uterus and placenta is a dynamic process, and the blood flow in a single vessel may change. Therefore, it could be difficult for the parameters of the uterine artery to represent the whole blood circulation state of the maternal-fetal interface in some cases. In the future studies, it is necessary to use standardized detection methods to evaluate the parameter selection and evaluation strategy of UAD in prospective researches with large sample sizes to obtain better prediction approaches. In addition, promoting the understanding of the etiology of pregnancy complications can help find novel detection methods and preventive measures.

## Author Contributions

YT and XY drafted the manuscript. XY conceived the idea and revised the manuscript. All authors read, contributed, and approved the final version.

## Conflict of Interest

The authors declare that the research was conducted in the absence of any commercial or financial relationships that could be construed as a potential conflict of interest.

## Publisher's Note

All claims expressed in this article are solely those of the authors and do not necessarily represent those of their affiliated organizations, or those of the publisher, the editors and the reviewers. Any product that may be evaluated in this article, or claim that may be made by its manufacturer, is not guaranteed or endorsed by the publisher.
